# Epidemiology of chronic dialysis treatment among children and adolescents in a capital of Northeastern Brazil

**DOI:** 10.1590/2175-8239-JBN-2025-0263en

**Published:** 2026-06-05

**Authors:** Ingrid Silva Medeiros, Janeide Leonar Alves Siqueira, Giselle Andrade Silva Lima Pires, Ana Lucia Guterres de Abreu Santos, Ricardo Ferreira Santos

**Affiliations:** 1 Universidade Federal do Maranhão, Hospital Universitário, Serviço de Nefrologia, Empresa Brasileira de Serviços Hospitalares, São Luís, MA, Brazil.; 2 Universidade Federal do Maranhão, Hospital Universitário, Pediatria, São Luís, MA, Brazil.

**Keywords:** Renal Insufficiency, Chronic, Renal Dialysis, Child, Adolescent

## Abstract

**Introduction:**

Information about the clinical and epidemiological profile of children and adolescents undergoing chronic dialysis in Brazil is limited.

**Objective:**

To describe the clinical and epidemiological characteristics of the pediatric population undergoing chronic outpatient dialysis treatment in a capital city in Northeastern Brazil.

**Methods:**

This is a descriptive, observational, and retrospective study whose population consisted of all children and adolescents undergoing chronic outpatient dialysis treatment in São Luís, capital of the state of Maranhão, Brazil, in 2023.

**Results:**

Thirty-two children and adolescents with chronic kidney disease (CKD) were undergoing dialysis treatment during this period, the majority (56.25%) being male. The mean age was 11.31 years (± 3.56). Regarding ethnicity, non-white individuals predominated (71.9%). The renal replacement therapy (RRT) modality was exclusively hemodialysis (HD), and the most commonly used vascular access at the end of the follow-up period was arteriovenous fistula (AVF) (56.2%). Regarding the underlying disease, 35% had an unknown etiology. There were nine transplants during the period, all from deceased donors. At that time, there was virtually universal access to erythropoiesis-stimulating agents and drugs for the prevention and treatment of bone mineral metabolic disorders.

**Conclusion:**

The high rate of unknown underlying disease and the exclusive use of HD as the RRT modality, rather than peritoneal dialysis, reflect diagnostic, therapeutic, and structural challenges. This scenario highlights the need for better planning of public policies aimed at managing pediatric CKD in the region.

## Introduction

Advanced chronic kidney disease (CKD) requiring renal replacement therapy (RRT) is a relatively rare condition among children and adolescents. In this age group, congenital anomalies of the kidneys and urinary tract (CAKUT), glomerulopathies, and genetic diseases are the most common causes of permanent functional loss^1^. In addition to transplantation, which would be the ideal option as it offers a better quality of life and longer survival, especially in this age group, other RRT modalities for CKD include hemodialysis (HD) and peritoneal dialysis (PD)^2^.

Information about the clinical and epidemiological profile of children and adolescents undergoing chronic dialysis in Brazil remains scarce and shows wide variation according to regional factors^3,4,5^. In this sense, the present work aims to describe the sociodemographic and clinical characteristics of the pediatric population undergoing outpatient RRT in the city of São Luís, capital of the state of Maranhão, located in the Northeast region of the country.

## Methods

This is a descriptive, retrospective, and observational study that analyzed the clinical and epidemiological profile of children and adolescents (<19 years) undergoing outpatient RRT in São Luís between January 1, 2023, and December 31, 2023.

In the capital of the state of Maranhão, the University Hospital of the *Universidade Federal do Maranhão* (HU-UFMA), part of the *Empresa Brasileira de Serviços Hospitalares* (EBSERH) network, centralizes the treatment and follow-up of children and adolescents requiring chronic RRT. Therefore, the data were obtained through the analysis of electronic medical records from the Nephrology Service and the dialysis unit of this institution.

The variables collected were sex, age, school attendance (attends regularly, attends irregularly, does not attend [dropout], does not attend [insufficient age]), ethnicity/race, and family socioeconomic level. Clinical variables included type of RRT (HD, PD), time on dialysis (in months), underlying disease, type of vascular access, use of erythropoietin (EPO) and drugs for the treatment and prevention of bone mineral metabolic disorders (BMD), registration on the waiting list, and transplants performed.

To investigate whether the pediatric dialysis population in this study met the recommended laboratory targets for calcium, phosphorus, parathyroid hormone (PTH), hemoglobin (Hb), transferrin saturation index (TSI), and ferritin levels for adequate control of BMD and anemia in CKD, the latest KDIGO (Kidney Disease: Improving Global Outcomes) recommendations for diagnosis, evaluation, and treatment of these conditions were used as parameters^6,7^. The following laboratory criteria were considered: PTH between 2 and 9 times the upper laboratory limit (normal value: 10–65 pg/mL; therefore, <585 pg/mL), calcium >8.8 mg/dL, phosphorus <5.5 mg/dL, Hb ≥11 g/dL, TSI ≥20%, and ferritin ≥100 ng/mL.

Information from electronic medical records was obtained in September and October 2024 and stored in a Microsoft Excel 2016 spreadsheet. Categorical variables were expressed as a percentage of the total, and continuous variables, after applying the Shapiro–Wilk test, were expressed as mean ± standard deviation for those with a normal distribution and as median (minimum and maximum values) for those with a non-normal distribution.

The study protocol was approved by the Research Ethics Committee, under number CAAE: 82641624.2.0000.5086. Informed consent was not required, as this is a retrospective study whose research instrument was the review of electronic medical records, and, therefore, there was no personal contact with the study population, nor diagnostic or therapeutic interventions.

## Results

Thirty-two children and adolescents underwent chronic dialysis treatment in São Luís, Maranhão, between January 1 and December 31, 2023. Of these, 56.25% (n=18) were male. The age of the study population ranged from 4 to 17 years, with a mean of 11.31 years (± 3.56). Twenty-eight (87.5%) patients were from inland areas of the state at the onset of RRT. Regarding ethnicity, 43.75% were of mixed race. As for school attendance, almost all (90.6%) were regular students. Regarding family income (FI), the majority (68.75%) had an income of up to one minimum wage (MW). Among the study population, during the evaluation period, all patients underwent conventional HD for four hours, three times a week, and none underwent PD. The dialysis time for each patient, calculated from the start of RRT until the transplantation date for the nine transplanted patients or until December 31, 2023, for the remaining individuals, ranged from 1 to 98 months, with a median of 26 months (Table 1).

**Table 1:** Demographic and social characteristics of 32 children and adolescents, who were on chronic dialysis in são luís, ma, in 2023

	Number of patients (n = 32)
Age (years): mean (± SD)^a^	11( ± 3.56)
Sex: n (%)	
Male	18 (56.25)
Female	14 (43.75)
Ethnicity/Race: n (%)	
Mixed	14 (43.75)
White	9 (28.12)
Black	6 (18.75)
Native American	3 (9.37)
School attendance: n (%)	
Regular	29 (90.62)
Irregular	1 (3.12)
Does not attend	1 (3.12)
Insufficient age	1 (3.12)
Family income: n (%)	
No income	2 (6.24)
Up to 1 MW^b^	22 (68.75)
1 to 2 MW	7 (21.87)
2 to 3 MW	1 (3.12)
Initial origin: n (%)	
State capital	4 (12.5)
Inland areas of the state	28 (87.5)
Time on dialysis (months): median	26 (1-98)^c^
Dialysis modality: n (%)	
Hemodialysis	32 (100)
Peritoneal dialysis	-

Abbreviations – ^a^SD: standard deviation;

^b^MW: minimum wage;

^c^(minimum–maximum).

**Figure 1:**
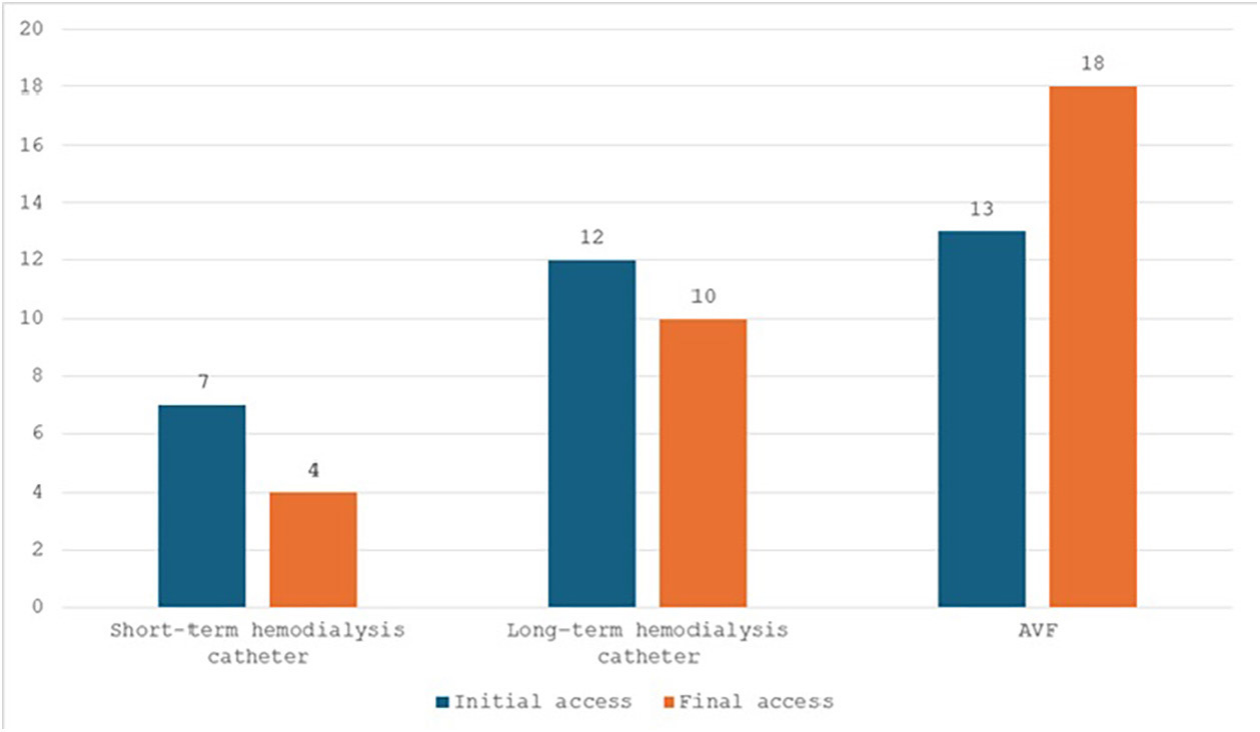
Vascular access at the beginning and end of follow-up among the 32 children and adolescents on a chronic dialysis program in São Luís, Maranhão in 2023.

Regarding vascular access for HD, at the beginning of the evaluation period, seven (21.9%) patients used short-term catheters, 12 (37.5%) used long-term catheters, and 13 (40.6%) had arteriovenous fistulas (AVF). The most prevalent access at the end of the study period, or until the transplant date in the case of the nine transplanted patients, was AVF (56.3%) (Figure 1).

**Figure 2:**
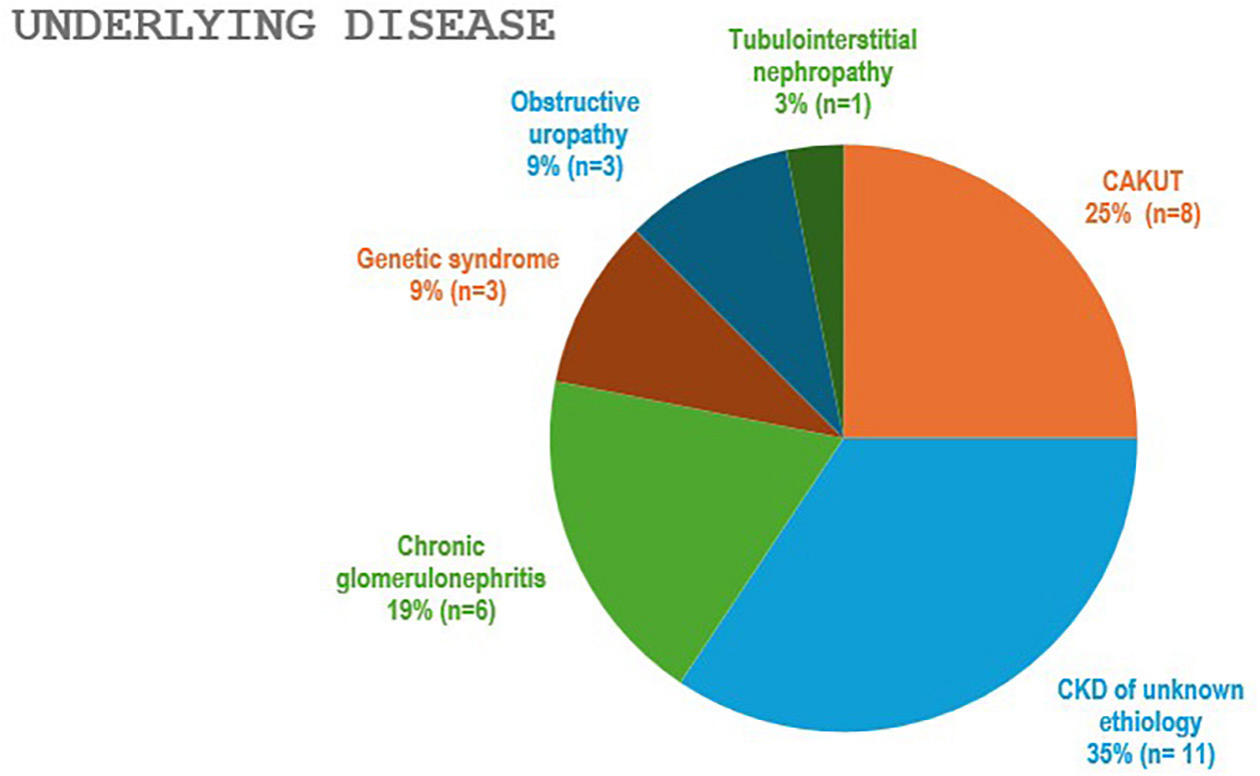
Underlying kidney disease among 32 children and adolescents on chronic dialysis in São Luís, Maranhão in 2023.

In the evaluated population, the underlying disease was not identified in 11 (35%) patients, and eight (25%) started dialysis due to CAKUT. The other causes are described in Figure 2.

Among the causes of CAKUT, posterior urethral valve (PUV) was the most prevalent, occurring in 5 of 8 (62.5%) patients. Among those attributed to chronic glomerulonephritis (CGN), half presented initially as nephrotic syndrome (3 of 6). One patient had diffuse mesangial sclerosis, one had lupus nephritis, one exhibited an inconclusive renal biopsy, and the remaining (3 of 6) had no histological examination. Of the genetic syndromes observed in three patients, one was Alport syndrome, another was autosomal recessive polycystic kidney disease, and the third was branchio-otorenal syndrome. Neurogenic bladder was the non-congenital obstructive uropathy observed in all three patients. The mean age among children undergoing RRT due to CGN was 11.5 (± 3.30) years, while the mean ages among those with genetic syndromes, CAKUTs, and non-congenital obstructive uropathy were 10 (± 5.35), 7.6 (± 2.97), and 16 (± 0.81) years, respectively.

**Figure 3:**
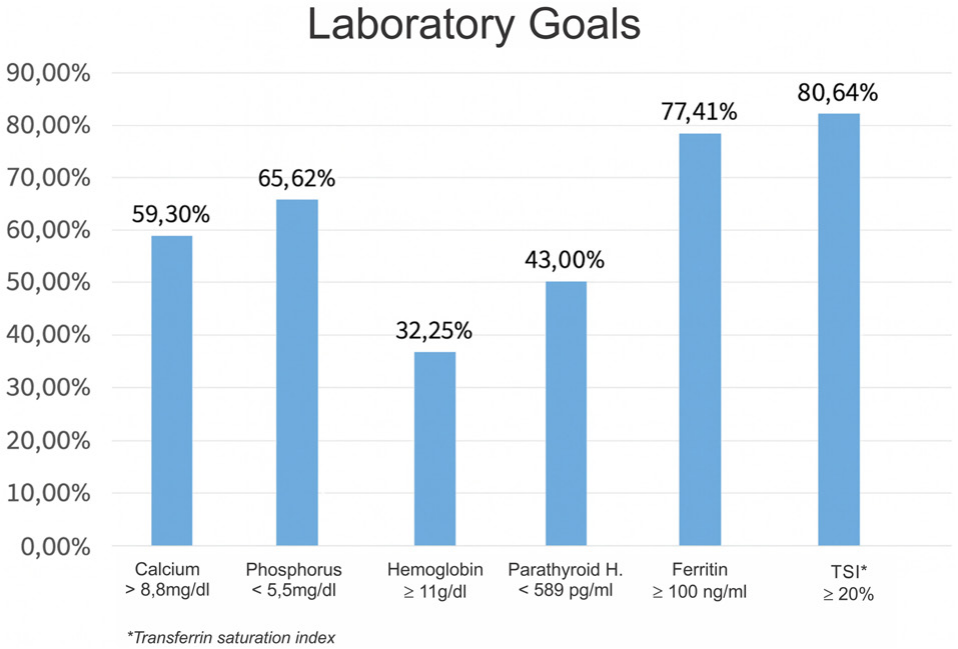
Proportion of patients who achieved laboratory targets for the control of bone mineral metabolic disorder and anemia among the 32 children and adolescents on a chronic dialysis program in São Luís, MA in 2023.

The majority (81.2%) of the children and adolescents evaluated (26 of 32) used EPO for the treatment of anemia in CKD. Regarding medications for the prevention or treatment of BMD, almost all (30 of 32) patients were receiving them. Of these, 12 (40%) used the combination of calcitriol and sevelamer; five (16.6%) calcitriol alone; three (10%) sevelamer alone; two (6.6%) sevelamer combined with cinacalcet; two (6.6%) calcium combined with calcitriol; two (6.6%) calcitriol, calcium, and sevelamer; two (6.6%) calcium alone; one (3.3%) the combination of sevelamer and calcium; and one (3.3%) paricalcitol alone. Regarding the targets adopted by the service for the laboratory values of calcium, phosphorus, PTH, Hb, ferritin, and TSI, the best results were observed for ferritin, with 80.6% of the studied population reaching the target in 2023 (Figure 3). The mean values at the end of the year for calcium (mg/dL), phosphorus (mg/dL), Hb (g/dL), and TSI (%) among the children and adolescents evaluated were 8.9 (± 0.68), 4.9 (± 1.05), 10.1 (± 1.63), and 30.78 (± 12.28), respectively. The median PTH and ferritin levels were 649.5 (121–2451) pg/mL and 329.91 (49.9–919.22) ng/mL, respectively.

**Figure 4:**
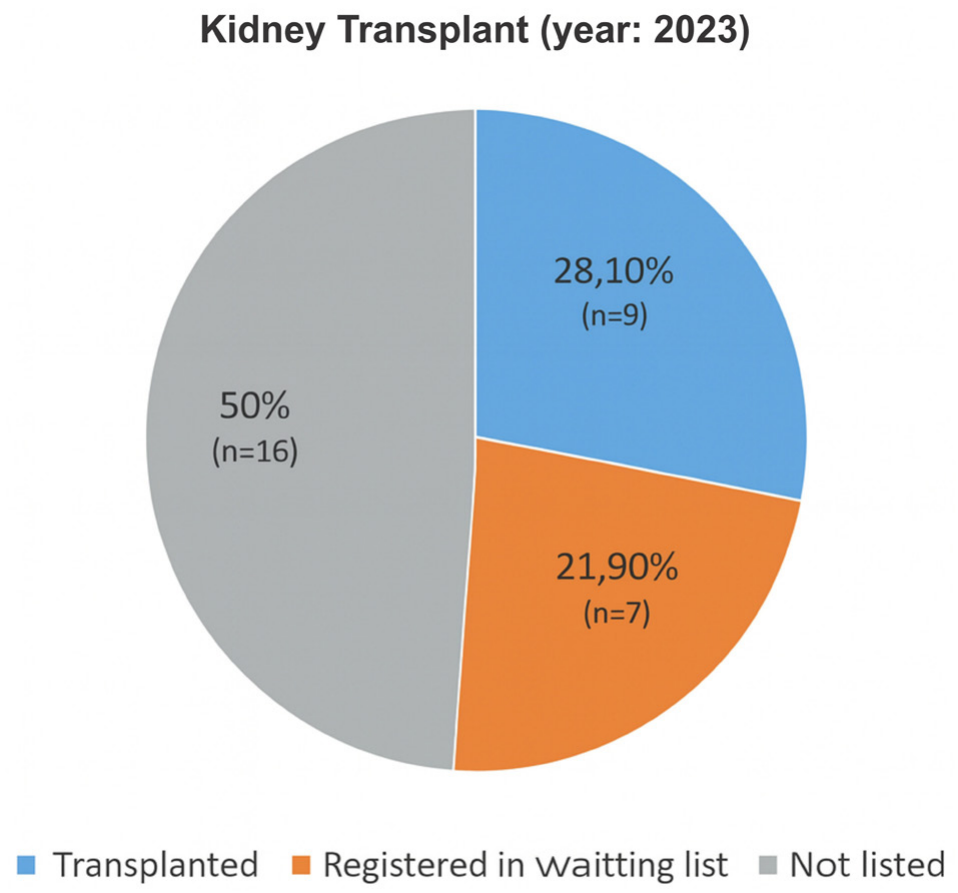
Transplant rate and waiting list registration for deceased donors among 32 children and adolescents on chronic dialysis in São Luís, MA in 2023.

During clinical follow-up, of the 32 children and adolescents, nine underwent transplantation during the study period, all with deceased donors. Of those who remained on hemodialysis (n = 24), seven (21.8%) were on the waiting list and 16 (50%) were not (Figure 4). Of these, 10 were eligible for transplantation and were only awaiting entry into the registration process. Of the remaining (6 of 16) patients, three were eligible but were not registered due to difficulty adhering to clinical treatment, and three had transient contraindications for surgery: one had recently undergone transplantation and experienced severe graft rejection; the second was awaiting urological surgical treatment (to resolve a case of PUV); and the third was not in a suitable clinical condition in 2023 due to severe heart failure with decompensated reduced ejection fraction.

## Discussion

The HU-UFMA, part of the EBSERH network, centralizes RRT for children and adolescents with chronic kidney disease in the state capital, São Luís. In this institution, during 2023, 32 pediatric patients were undergoing chronic dialysis treatment.

Considering the demographic census of the previous year carried out by the *Instituto Brasileiro de Geografia e Estatística* (IBGE)^8^, the municipality had 1,037,775 inhabitants. Of these, 305,775 were 18 years of age or younger, corresponding to a local prevalence of approximately 104 pediatric patients on chronic dialysis per million age-related population (pmarp). Konstantyner et al.^3^ specifically evaluated the epidemiology of chronic dialysis treatment among children and adolescents in Brazil in 2011 and found a national prevalence of 20/pmarp, ranging from 13.8 in the Northeast region to 27.7 in the South region of the country.

Similarly to what is observed across different regions of our country, there is a wide global geographic variation in the prevalence of chronic kidney disease requiring dialysis (CKD-d) among patients <19 years of age, which may be linked to disparities in resources and trained personnel to care for this population. For example, in 2008, 80% of children and adolescents with CKD-d were concentrated in Europe, Japan, the United States (USA), and Canada. Prevalence in different countries at the end of this year ranged from 18/pmarp in Russia, through 65/pmarp in Canada and Australia, 85/pmarp in the USA, reaching up to approximately 100/pmarp in Finland, where the high incidence of CKD-d in newborns, caused by Finnish-type nephrotic syndrome, is responsible for this high rate^9^.

More recent data show that in Brazil, in July 2023, there were 157,357 patients on dialysis, and 0.9% were under 20 years of age, which corresponded to approximately 1,416 patients in this age group^10^. The overall prevalence was 771 per million population, while among children and adolescents, including those up to age 19, it was approximately 35/pmarp.

According to data from the Brazilian Society of Nephrology (SBN), Maranhão regional chapter, with the exception of Imperatriz, the state’s second-most populous city, located about 630 km from the capital, no other unit in inland areas of the state provides care for children (<11 years) with CKD-d. This may explain the high rate observed in the capital, as described in this study. Far from signifying an outbreak of chronic kidney disease in the pediatric population of São Luís or representing an actual prevalence, this finding reflects the migration of patients and families to the capital in search of specialized care, which is not available in their region of origin. Of the 32 patients on RRT evaluated in 2023, only four were residents of São Luís, which would represent a true prevalence of about 13/pmarp, very similar to that identified by other authors in the Northeast region of the country^3^.

Among the population described in this study, the majority were male (56.3%), as previously demonstrated in other epidemiological studies addressing the same age group, which was not unexpected^3,7^. Some congenital anomalies of the urinary tract have a higher prevalence in boys, such as renal dysplasia and stenosis of the ureteropelvic junction obstruction, in addition to PUV, which is exclusive to males^1^.

The mean age of the studied population was approximately 11 years, approaching that of other national studies that also evaluated chronic dialysis treatment among children and adolescents^3,4,5^. In the USA and Western Europe, the incidence of CKD-d is twice as high in adolescents (11–19 years) as in children (≤10 years)^9^. Since this study assessed only the prevalence of this condition, it was not possible to determine which pediatric age group had the highest incidence in our setting, considering that the mean dialysis time in the evaluated group was 2 years and 7 months, ranging from 2 weeks to 98 months.

Regarding ethnicity, the majority (71.8%) were declared as non-white in the medical records reviewed. In the USA, African American children have twice the risk of CKD-d compared with white children^11^. In Australia and New Zealand, the overall incidence of kidney disease is higher among Aboriginal people than among the rest of the population in the pediatric age range, although the incidence of dialysis is higher only among those over the age of 15. In the United Kingdom, in 2008, the prevalence and incidence of this condition detected among children who emigrated from Southeast Asia were 2.5 and 1.5 times higher, respectively, than in the white population aged 0 to 15 years. One possible explanation for these findings is that the social and economic inequalities observed between these ethnic groups may increase their biological vulnerability, in addition to potentially limiting access to early diagnosis and treatment.

In Brazil, on the other hand, Konstantyner et al.^3^ noted that the South and Southeast regions had the highest prevalence and incidence of CKD-d, which contradicts the expected result, since these are the regions with the highest prevalence of white children on RRT^3^. More than social or biological factors, this finding is mainly associated with the racial characteristics of this territorial area, colonized throughout the last century by a considerable population of European immigrants and characterized by a lower proportion of non-white individuals, according to IBGE^8^. Furthermore, in the same multicenter study cited, there was a higher prevalence of non-whites in the Northeast, North, and Central-West regions, territorial areas with lower incidence and prevalence of CKD, which apparently reflects only the racial diversification of the pediatric population on chronic dialysis according to regional factors, rather than a cause-and-effect relationship. In the state of Maranhão, for example, 79% of the population self-identifies as mixed-race, Black, or Native American, and only 20.1% consider themselves White^8^. These data are very close to those found in the present study regarding the pediatric population on dialysis.

When assessing the school attendance of children and adolescents undergoing dialysis treatment in São Luís, the majority were regular attendees and in the appropriate grade (90.6%). These findings do not correspond to those reported by other authors in other institutions, who identified a high proportion of this population being absent from school, with academic delays or excessive absences^12,13,14^. The motivations are diverse. Fatigue and pain restrict daily activities, in addition to frequent hospitalizations and medical appointments, which have a cumulative effect on their time, monopolizing daily routines and limiting their ability to attend school and participate in extracurricular activities. This contributes to poorer academic performance, social isolation, low self-esteem, and lower educational attainment and employability in adulthood^12^.

This positive difference described in the present study is attributed to the presence of an educational project at a local municipal school, implemented through an agreement between HU-UFMA and the state capital’s Department of Education, which provides educational support for basic subjects during treatment hours. This initiative is already well-established among children undergoing cancer treatment in Brazil but is considered uncommon among those undergoing RRT.

More than half of the patients (68.75%) evaluated in this study had a FI of up to one MW. Two families had no income in 2023, as the mother and child were staying at a support facility from their municipality, located in the capital, and did not yet have access to the continuous cash benefit provided by the Federal Government. Only one family had a FI between two and three MW. Other national studies have reported prevalences ranging from approximately 39% to 87% of FI below one MW among children and adolescents on dialysis^15,16^.

The data presented here therefore demonstrate that the majority of patients treated in the capital belong to low-income families, reflecting the socioeconomic characteristics of the population served, most of whom come from other municipalities within the state, which indicates high financial vulnerability and total dependence on government support to carry out their treatment. Low family income also highlights limitations in access to additional resources that could optimize their care.

Among the studied population, all underwent HD as the RRT modality during the evaluated period, since the HU-UFMA has not performed PD since 2020. The exclusive use of HD as RRT in this population sample, from a reference hospital for pediatric dialysis in the state, raises questions about the reasons for the lack of availability of the peritoneal method in the region and highlights the need to expand access to this therapeutic option, which is considered more suitable for pediatric management. Significant advantages for this age group include greater preservation of residual renal function and vascular access for future transplantation; less impact on growth retardation and the need for transfusions; absence of painful punctures; superior efficiency of the method in children compared to adults due to their larger peritoneal surface area relative to body surface area; and the possibility of performing RRT in a home setting, promoting better quality of life and greater adherence to treatment^2^.

For this reason, therefore, PD constitutes a viable option for patients residing in inland areas of the state, far from specialized centers, as it avoids long commutes and changes of residence, as observed in the population evaluated in this study. In addition, newborns and infants who are only a few months old or underweight, extremely premature infants, and those born with congenital anomalies of the kidneys and urinary tract leading to CKD have in this method their only possibility of survival until an eventual transplant^2^.

In a study sponsored by the SBN, Palma et al.^17^ evaluated 60 pediatric dialysis centers in the country and identified that 23 of them no longer offered PD. Of the remaining 37, 19 had to convert from PD to HD due to a shortage of supplies. The authors argue that the lower availability of PD in Brazil is related to the low prices paid to supply companies, lower remuneration of the professionals involved, and the lack of qualified professionals and replacement materials, which has resulted in the preference for HD, even in children with an indication for PD^17^.

The data presented here demonstrated that the mean time on dialysis per patient, measured from the start of RRT until the date of transplantation for the nine patients transplanted in 2023, or until December 31 of the same year for those who remained on dialysis, was approximately 31 months (2 years and 7 months), with a median of 26 months (2 years and 2 months). In Switzerland, this variable decreased from 1.6 years in the 1980s to 0.34 years between 2010 and 2015^18^. In Brazil, a prevalence study conducted in Minas Gerais in 2006 showed a mean duration of pediatric RRT of 2.8 years (± 2.2 years)^19^, similar to the case series described here.

The shorter waiting time for transplantation reported in epidemiological surveys from other countries may reflect better organ allocation policies for this group of patients. This disparity may also be related to differences in CKD-d management protocols across regions and the greater availability of donors, which directly impact the time that children and adolescents remain on RRT. Among the 32 children and adolescents studied who were on dialysis, there were nine kidney transplants in 2023 (28.1%), all with deceased donors. Among those who did not undergo transplantation, seven were listed on the waiting list, whereas 16 were not. Of these, most had an indication for this treatment modality and were merely awaiting entry into the listing process, which likely indicates the need to develop strategies within the service to better direct patients toward faster access to the single waiting list.

The data presented here also showed that at the end of 2023, or up to the date of transplantation among those who underwent the procedure, the predominant vascular access was AVF (56.3%), followed by long-term (31.2%), and short-term (12.5%) HD catheters.

In the recent past, perhaps due to the ease and convenience of central venous catheters (CVCs), AVFs were not widely used in children. Of the 870 accesses placed in 552 patients enrolled in the International Pediatric Hemodialysis Network (IPHN) Registry, approximately 72% were CVCs. Technical difficulties in creation and maturation, inexperience of pediatric dialysis nurses in puncturing small-caliber arterialized veins, and parental concern regarding pain resulted in reluctance to consider AVFs as the first option^20,21^. Evidence of the benefits of this type of access compared with CVCs in adult dialysis programs has been known for some time. More recent studies in the pediatric age group have also demonstrated similar results, including more efficient dialysis, a lower infection rate and fewer access changes, and, consequently, lower hospitalization rates^20,21^.

Regarding vascular access, data from the United States Renal Data System (USRDS) showed a 76% rate of CVC use in the pediatric RRT population in that country in 2024^22^, almost double the rates observed in São Luís. These results could likely be explained by a higher likelihood of short-term kidney transplantation among North American children than in the population evaluated here, prioritizing the creation of AVFs in that country only for those who, for some reason, did not have a defined timeframe for discontinuing hemodialysis. Contrasting this hypothesis, a publication from the same year that evaluated data from the Global Kidney Health Atlas of the International Society of Nephrology (ISN-GKHA) reported that in approximately 25% of the centers evaluated, more than half of the patients initiated hemodialysis with AVFs^23^, especially in developed countries, where children on dialysis traditionally have a shorter waiting time for kidney transplantation, similar to what occurs in the USA.

Approximately one-third of the children evaluated here did not have an identified CKD etiology, a finding similar to that of the national epidemiological survey conducted in 2011 by Konstantyner et al.^3^, whose results were influenced, with regard to this variable, by the high rates of non-diagnosis of the underlying disease in the pediatric population of the North and Northeast regions during that period^3^. Late diagnosis of the disease, attributed to difficulty accessing large centers and specialized professionals for the diagnosis and management of kidney disease in childhood, may be responsible for these findings. For example, among the pediatric dialysis population in São Luís in 2023, as already described, almost 90% came from inland areas of the state, which has one of the lowest doctor-to-patient ratios and Human Development Index in the country^24^.

In São Luís, CAKUTs accounted for 25% of cases, with PUV being the most frequent. Data from the North American Pediatric Renal Trials and Collaborative Studies (NAPRTCS), which receives pediatric nephrology data from over 7,000 children and adolescents in the USA, identified the group formed by CAKUTs (48%) and hereditary nephropathies (10%) as the most common etiologies, followed by CGNs (14%). In this registry, as expected, the distribution of causes varied with age. CAKUTs predominated among younger children, just as CGNs predominated among adolescents^25^. Similarly, among children on dialysis in São Luís in 2023, the mean age of those with CAKUTs was 7.6 years (± 2.97), while among those diagnosed with CGNs it was 11.5 (± 3.3).

Similar data have been described in the European population, based on epidemiological surveys from Italy and Belgium. The proportions of CAKUTs (58–59%) and hereditary nephropathies (15–19%) were slightly higher, while those of CGNs were slightly lower (5–7%), when compared with the NAPRTCS database^26,27^. On the other hand, a high prevalence of genetic diseases has been reported in the Middle East, which is attributed to high rates of consanguineous marriages. Similarly, CGNs are the main cause of CKD-d in the pediatric age group in several studies involving children from India, Southeast Asia, and Sub-Saharan Africa, with a prevalence ranging between 30% and 60%, findings that may be related to the high prevalence of viral, bacterial, and parasitic infections in those regions^9^. In these specific settings, it can also be speculated that the anticipated difficulty young children face in accessing RRT, with consequent early mortality, could skew the statistics toward CGNs, which characteristically affects older pediatric age groups.

The data presented here also demonstrated near-universal access for children and adolescents undergoing dialysis in the capital to erythropoiesis-stimulating agents and medications for the prevention and treatment of BMD, all provided free of charge by the Unified Health System (SUS) in Brazil. After completing prescriptions, reports, and consent forms, the pediatric nephrology service at HU-UFMA/EBSERH, through its pharmacy, retrieves the medications and provides them to families for home use in the management of BMD. In addition, the service provides for hospital storage and administration of EPO for the treatment of anemia.

Despite the ease of obtaining the aforementioned medications, only 43% of the children evaluated in this study reached the PTH target (<589 pg/mL) recommended by KDIGO 2017^7^. The median level of this hormone among the 32 children and adolescents evaluated here was approximately 650 pg/mL. For comparison, in the last Brazilian Dialysis Census of 2023, which covered a predominantly adult population, a percentage approximately twice as high (81%) of patients reached the PTH target (<600 pg/mL) among the clinics that submitted data for publication^10^.

Borzych and Rees^28^, in turn, when evaluating data from 890 children and adolescents on peritoneal dialysis from 24 countries, found that the majority (70%) had PTH above the adopted target (<300 pg/mL), using the recommendations of the KDOQI (Kidney Disease Outcomes Quality Initiative) of 2005^28,29^ as a parameter. Low serum calcium and bicarbonate levels, elevated phosphorus levels, female sex, and longer time on dialysis were independent risk factors for poor control of hyperparathyroidism in that population^28^. Other studies have also identified tubulointerstitial kidney diseases and 25-OH vitamin D deficiency as variables related to the most severe forms of this type of bone mineral disorder in this age group^29^. Schramm et al.^4^, when evaluating 35 children and adolescents on dialysis in Manaus, capital of Amazonas, Brazil, between June 2018 and April 2019, observed hypocalcemia in 82.8% of patients, hyperphosphatemia in 57.2%, and PTH values above the target in 48.6%.

In the present study, contrary to what might be expected given the high PTH values observed outside the target range, most children and adolescents had mean calcium and phosphorus levels within the recommended range. Regarding 25-OH vitamin D and bicarbonate levels, in 2023, when the study was conducted, regular measurement was not yet routine in the service, making it impossible to determine whether there was any impact on the results described here.

It remains uncertain whether hyperparathyroidism in CKD is more difficult to control in children and adolescents than in adults, but it is particularly concerning due to its irreversible impact on growth and bone development in the pediatric population.

The majority (81.2%) of the children and adolescents evaluated (26 of 32) were receiving EPO for the treatment of CKD-related anemia; however, only 32% achieved the expected average target (Hb ≥11 g/dL) by the end of the study period. This rate was lower than that observed in the latest Brazilian Dialysis Census of the SBN (70%). It should be noted that in this case series, an Hb threshold of >10 g/dL was used as a parameter, and almost all patients were adults^10^. In the state of Amazonas, in the aforementioned study by Schramm et al.^4^, anemia was observed in 80% of children and adolescents, and all were using EPO. Ferritin and TSI values were outside the expected range in only 26% and 28%, respectively. Absolute iron deficiency was identified in 17% of patients and functional iron deficiency in 11%, in the same publication^4^. Khalil et al.^30^, in an 18-month follow-up of a cohort of 78 children and adolescents on hemodialysis, observed that, at the end of the follow-up period, only 29.3% of patients had reached the Hb target, 50% the TSI target, and 11.9% the ferritin target, as recommended by KDIGO^30^.

In the present study, 80.6% and 77.4%, respectively, of the pediatric dialysis population evaluated achieved the recommended annual average for ferritin and TSI values, the main variables related to an inadequate response to EPO^6^. However, it is worth noting that, in addition to absolute or relative iron deficiency, other factors may be associated with a poor response to erythropoiesis-stimulating agents in this population, such as malignancy, infections, inflammation, vitamin deficiencies, hyperparathyroidism, and inadequate dialysis^31^. Not all of these variables were explored in the present study, but they may have contributed to the low Hb target achievement observed in the case series presented here.

## Conclusions

Thus, based on the results presented here, it can be concluded that the population of children and adolescents undergoing dialysis in São Luís, the capital of the state of Maranhão, in 2023, had a mean age of 11 years and approximately 2 years and 7 months on RRT. They were predominantly non-white, from low-income families, with a slight majority of males, and more than 90% were from inland areas of the state. All were treated with HD, since PD was not available at HU-UFMA, the hospital that centralized pediatric RRT in the capital. At the end of the study period, most had AVF access, maintained regular school attendance with minimal absenteeism, and had almost universal access to medications for the treatment and prevention of anemia and BMD. Despite this, less than half achieved the target range for PTH and Hb. More than a third of the population evaluated did not have an identified underlying disease, which may reflect a lack of prior follow-up, probably due to difficulty accessing a specialist before the onset of RRT. A significant number of children and adolescents underwent transplantation during the evaluated period; however, among those who did not, more than half had the clinical and social conditions to do so but, for some reason, were not yet registered on a waiting list. This demonstrates the need to implement improvements in access to this alternative form of RRT, which is recognized as offering greater survival and better quality of life, especially in this age group.

## Data Availability

The entire dataset supporting the results of this study is available upon request to the corresponding author [Ricardo Ferreira Santos, ricardonefro12@hotmail.com]. The dataset is not publicly available because it contains information that compromises the privacy of the research participants.
