# Supplementary material for: Epidemiology of chronic dialysis treatment among children and adolescents in a capital of Northeastern Brazil
Source: J Bras Nefrol. 2026 Jun 5;48(3):e20250263. doi: 10.1590/2175-8239-JBN-2025-0263en (PMC13258131; doi:10.1590/2175-8239-JBN-2025-0263en)
Supplement: Translation

# Translation: Epidemiologia do tratamento dialítico crônico entre crianças e adolescentes em uma capital do Nordeste do Brasil

**DOI:** 10.1590/2175-8239-JBN-2025-0263pt

**Authors:** Ingrid Silva Medeiros, Janeide Leonar Alves Siqueira, Giselle Andrade Silva Lima Pires, Ana Lucia Guterres de Abreu Santos, Ricardo Ferreira Santos

**Affiliations:** 1 Universidade Federal do Maranhão, Hospital Universitário, Serviço de Nefrologia, Empresa Brasileira de Serviços Hospitalares, São Luís, MA, Brasil.; 2 Universidade Federal do Maranhão, Hospital Universitário, Pediatria, São Luís, MA, Brasil.

**Keywords:** Insuficiência Renal Crônica, Diálise Renal, Criança, Adolescente

## Abstract

**Introdução:**

Informações acerca do perfil clínico e epidemiológico de crianças e adolescentes submetidos à diálise crônica no Brasil são escassas.

**Objetivo:**

Descrever as características clínicas e epidemiológicas da população pediátrica em tratamento dialítico crônico ambulatorial em uma capital do Nordeste do Brasil.

**Métodos:**

Trata-se de um estudo descritivo, observacional e retrospectivo, cuja população foi composta por todas as crianças e adolescentes em tratamento dialítico crônico ambulatorial em São Luís, capital do estado do Maranhão – Brasil, no ano de 2023.

**Resultados:**

Trinta e duas crianças e adolescentes portadores de doença renal crônica (DRC) estiveram em tratamento dialítico neste período, sendo a maioria (56,25%) do sexo masculino. A idade média foi de 11,31 anos (± 3,56). Quanto à etnia, predominavam os não brancos (71,9%). O tipo de terapia renal substitutiva (TRS) foi exclusivamente hemodiálise (HD), e o acesso vascular mais utilizado no final do período de seguimento foi a fístula arteriovenosa (56,2%). Acerca da doença de base, 35% não tiveram a etiologia identificada. Houve nove transplantes no período, todos com doador falecido, e, à época, havia acesso praticamente universal aos agentes estimuladores da eritropoiese e às drogas para prevenção e tratamento dos distúrbios do metabolismo mineral ósseo.

**Conclusão:**

A elevada taxa de desconhecimento da doença de base e a HD como método exclusivo de TRS, em detrimento da diálise peritoneal, refletem desafios diagnósticos, terapêuticos e estruturais, sublinhando a necessidade de melhor planejamento das políticas públicas voltadas ao manejo da DRC pediátrica na região.

## Introdução

A doença renal crônica (DRC) em estágio avançado, com necessidade de terapia renal substitutiva (TRS), é uma condição relativamente rara entre crianças e adolescentes. Nesta faixa etária, as anomalias congênitas dos rins e das vias urinárias (*Congenital Anomalies of the Kidneys and Urinary Tracts* – CAKUTs), as glomerulopatias e as doenças genéticas são as causas mais comuns de perda funcional definitiva^1^. Além do transplante, que seria a opção ideal por oferecer melhor qualidade de vida e maior sobrevida, especialmente nessa faixa etária, as outras modalidades de TRS na DRC incluem a hemodiálise (HD) e a diálise peritoneal (DP)^2^.

Informações acerca do perfil clínico e epidemiológico de crianças e adolescentes submetidos à diálise crônica no Brasil ainda são escassas e apresentam ampla diversificação de acordo com fatores regionais^3,4,5^. Nesse sentido, o presente trabalho objetiva apresentar as características sociodemográficas e clínicas da população pediátrica em TRS ambulatorial na cidade de São Luís, capital do estado do Maranhão, situada na região Nordeste do país.

## Métodos

Trata-se de um estudo descritivo, retrospectivo e observacional, que analisou o perfil clínico e epidemiológico de crianças e adolescentes (<19 anos) em TRS ambulatorial em São Luís, no período entre 01/01/2023 e 31/12/2023.

Na capital do estado do Maranhão, o Hospital Universitário da Universidade Federal do Maranhão (HU-UFMA), da rede da Empresa Brasileira de Serviços Hospitalares (EBSERH), centraliza o tratamento e seguimento de crianças e adolescentes que necessitam de TRS crônica. Dessa forma, os dados foram obtidos a partir da análise dos prontuários eletrônicos do Serviço de Nefrologia e da unidade de diálise dessa instituição.

As variáveis coletadas foram: sexo, idade, frequência escolar (frequenta regularmente, frequenta irregularmente, não frequenta [desistência], não frequenta [idade insuficiente]), etnia/cor, nível socioeconômico familiar, tipo de TRS (HD, DP), tempo em diálise (em meses), doença de base, tipo de acesso vascular, uso de eritropoietina (EPO) e de drogas para tratamento e prevenção dos distúrbios do metabolismo mineral ósseo (DMO), inscrição em fila e transplantes realizados.

Para investigar se a população pediátrica dialítica do presente estudo atingiu as metas laboratoriais recomendadas para os níveis de cálcio, fósforo, paratormônio (PTH), hemoglobina (Hb), índice de saturação de transferrina (ISAT) e ferritina, para o controle adequado da DMO e da anemia na DRC, utilizaram-se como parâmetros as recomendações mais recentes do KDIGO (*Kidney Disease: Improving Global Outcomes*) para diagnóstico, avaliação e tratamento dessas condições^6,7^. Foram considerados os seguintes critérios laboratoriais: PTH entre 2 e 9 vezes o limite superior do valor de referência laboratorial (valor normal: 10–65 pg/mL; portanto <585 pg/mL), cálcio >8,8 mg/dL, fósforo <5,5 mg/dL, Hb ≥11 g/dL, ISAT ≥20% e ferritina ≥100 ng/mL.

As informações dos prontuários eletrônicos foram obtidas nos meses de setembro e outubro de 2024 e armazenadas em uma planilha do Microsoft Excel 2016. As variáveis categóricas foram expressas em porcentagem do total, e as contínuas, após aplicação do teste de *Shapiro-Wilk*, em média ± desvio padrão para as que apresentavam distribuição normal e em mediana (valores mínimo e máximo) para aquelas com distribuição não normal.

O protocolo do estudo foi aprovado pelo Comitê de Ética em Pesquisa, sob o número CAAE: 82641624.2.0000.5086. Não houve necessidade de termo de consentimento informado, pois trata-se de um estudo retrospectivo, cujo instrumento de pesquisa foi a revisão de prontuários eletrônicos, e não houve, portanto, contato pessoal com a população estudada, nem intervenções diagnósticas ou terapêuticas.

## Resultados

Trinta e duas crianças e adolescentes estiveram em tratamento dialítico crônico em São Luís, MA, entre 01 de janeiro e 31 de dezembro de 2023. Destas, 56,25% (n = 18) eram do sexo masculino. A idade da população estudada variou entre 4 e 17 anos, com média de 11,31 anos (± 3,56). Vinte e oito (87,5%) eram procedentes do interior do estado no início da TRS. Em relação à etnia, 43,75% eram pardos. Quanto à frequência escolar da amostra, a quase totalidade (90,6%) era regular. No que diz respeito à renda familiar (RF), a maioria (68,75%) dispunha de até um salário mínimo (SM). Entre a população estudada, no período avaliado, todos realizavam HD convencional por quatro horas, três vezes por semana, e nenhum realizava DP. O tempo em diálise de cada paciente, contado do início da TRS até a data do transplante, no caso dos nove pacientes transplantados, ou até 31 de dezembro de 2023, no caso dos demais, variou de 1 a 98 meses, com mediana de 26 meses (Tabela 1).

**Tabela 1:** Características demográficas e sociais de 32 crianças e adolescentes que estiveram em programa de diálise crônica em são luís, ma, no ano de 2023

	N° de pacientes (n = 32)
Idade (anos): media (± DP)^a^	11 (± 3,56)
Sexo: n (%)	
Masculino	18 (56,25)
Feminino	14 (43,75)
Etnia/Raga: n (%)	
Parda	14 (43,75)
Branca	9 (28,12)
Preta	6 (18,75)
Indígena	3 (9,37)
Frequencia escolar: n (%)	
Regular	29 (90,62)
Irregular	1 (3,12)
Ausencia	1 (3,12)
Idade insuficiente	1 (3,12)
Renda familiar: n (%)	
Sem renda	2 (6,24)
Ate 1 SM^b^	22 (68,75)
1 a 2 SM	7 (21,87)
2 a 3 SM	1 (3,12)
Procedencia inicial: n (%)	
Capital	4 (12,5)
Interior do estado	28 (87,5)
Tempo em dialise (meses): medialna	26 (1-98)^c^
Modalidade: n (%)	
Hemodialise	32 (100)
Dialise peritoneal	-

Abreviaturas – ^a^DP: desvio padrão;

^b^SM: salário mínimo;

^c^(mínimo – máximo).

Em relação ao acesso vascular para realização da HD, no início do período de avaliação, sete (21,9%) utilizavam cateter de curta permanência, 12 (37,5%) de longa permanência e 13 (40,6%) fístula arteriovenosa (FAV). O acesso mais prevalente no final do período da pesquisa (31/12/2023) ou até a data do transplante, no caso dos nove pacientes transplantados, foi a FAV (56,3%) (Figura 1).

**Figura 1:** Acesso vascular no início e ao final do seguimento entre as 32 crianças e adolescentes que estiveram em programa de diálise crônica em São Luís, MA, no ano de 2023.

Na população avaliada, a doença de base não foi identificada em 11 (35%) pacientes, e oito (25%) iniciaram diálise devido a CAKUTs. As demais causas estão descritas na Figura 2.

**Figura 2:** Doença renal de base entre 32 crianças e adolescentes que estiveram em programa de diálise crônica em São Luís, MA, no ano de 2023.

Dentre as causas de CAKUT, a válvula de uretra posterior (VUP) foi a mais prevalente, ocorrendo em 5/8 (62,5%) pacientes. Entre aquelas atribuídas à glomerulonefrite crônica (GNC), em metade a apresentação inicial foi como síndrome (sd.) nefrótica (3/6), sendo um paciente com esclerose mesangial difusa, um com nefrite lúpica, um com biópsia renal inconclusiva e os demais (3/6) sem estudo histológico. Das sd. genéticas observadas em três pacientes, uma foi a sd. de *Alport*, outra a doença renal policística autossômica recessiva e a terceira a sd. brânquio-oto-renal. Bexiga neurogênica foi a uropatia obstrutiva não congênita observada em todos os três pacientes identificados. A média de idade entre as crianças em TRS devido à GNC foi de 11,5 (± 3,30) anos, enquanto entre os portadores de sd. genéticas, CAKUTs e uropatia obstrutiva não congênita foi de 10 (± 5,35), 7,6 (± 2,97) e 16 (± 0,81) anos, respectivamente.

A maioria (81,2%) das crianças e adolescentes avaliados (26/32) utilizava EPO para tratamento da anemia da DRC. Quanto aos medicamentos para prevenção ou tratamento da DMO, quase todos (30/32) faziam uso. Destes, 12 (40%) utilizavam a associação entre calcitriol e sevelamer; cinco (16,6%) calcitriol isoladamente; três (10%) apenas sevelamer; dois (6,6%) sevelamer associado ao cinacalcete; dois (6,6%) cálcio juntamente com calcitriol; dois (6,6%) calcitriol, cálcio e sevelamer; dois (6,6%) somente cálcio; um (3,3%) a associação de sevelamer e cálcio; e um (3,3%) apenas paricalcitol. Em relação às metas adotadas pelo serviço para os valores laboratoriais de cálcio, fósforo, PTH, Hb, ferritina e ISAT, os melhores resultados foram observados para a ferritina, com 80,6% da população estudada atingindo o objetivo no ano de 2023 (Figura 3). Os valores médios ao final do ano de cálcio (mg/dL), fósforo (mg/dL), Hb (g/dL) e ISAT (%) entre as crianças e adolescentes avaliados foram, respectivamente, 8,9 (± 0,68), 4,9 (± 1,05), 10,1 (± 1,63) e 30,78 (± 12,28). A mediana do PTH e da ferritina foi, respectivamente, 649,5 (121–2451) pg/mL e 329,91 (49,9–919,22) ng/mL.

**Figura 3:** Proporção de pacientes que atingiram as metas laboratoriais para o controle do distúrbio do metabolismo mineral ósseo e da anemia entre as 32 crianças e adolescentes que estiveram em programa de diálise crônica em São Luís, MA, no ano de 2023.

Durante o acompanhamento clínico das 32 crianças e adolescentes, nove transplantaram no período do estudo (ano de 2023), todos com doadores falecidos. Entre aquelas que permaneceram em hemodiálise (n = 24), sete (21,8%) estavam listadas em fila, e 16 (50%) não (Figura 4). Destes, 10 possuíam indicação para transplante e estavam aguardando apenas a entrada no processo de inscrição. Dos demais (6/16), três apresentavam indicação, mas não estavam inscritos devido à dificuldade de adesão ao tratamento clínico, e três apresentavam contraindicações transitórias para a cirurgia: um havia sido transplantado recentemente e apresentou rejeição grave do enxerto; o segundo apresentava pendência de tratamento cirúrgico urológico (para resolução do quadro de VUP); e o terceiro não possuía condições clínicas no ano de 2023 devido à insuficiência cardíaca grave com fração de ejeção reduzida descompensada.

**Figura 4:** Taxa de transplantes e dados de inscrição em fila de espera para doador falecido entre 32 crianças e adolescentes que estiveram em programa de diálise crônica em São Luís, MA, no ano de 2023.

## Discussão

O HU-UFMA, da rede EBSERH, centraliza a TRS de crianças e adolescentes com doença renal crônica na capital do estado, São Luís. Nessa instituição, durante o ano de 2023, 32 pacientes pediátricos estiveram em tratamento dialítico crônico.

Considerando-se o censo demográfico do ano anterior, realizado pelo Instituto Brasileiro de Geografia e Estatística (IBGE)^8^, o município possuía 1.037.775 habitantes; destes, 305.775 tinham idade igual ou menor a 18 anos, o que corresponde a uma prevalência local de cerca de 104 pacientes pediátricos em diálise crônica/milhão de população da mesma faixa etária (pmpmfe). Konstantyner et al.^3^ avaliaram especificamente a epidemiologia do tratamento dialítico crônico entre crianças e adolescentes no Brasil, no ano de 2011, e encontraram uma prevalência nacional de 20/pmpmfe, variando de 13,8 na região Nordeste a 27,7 na região Sul do país.

Tal como ocorre entre as diferentes regiões do nosso país, existe uma ampla variação geográfica mundial na prevalência de doença renal crônica dialítica (DRC-d) entre pacientes <19 anos, o que pode estar ligado à disparidade de recursos e de pessoal treinado para atendimento desta população. Por exemplo, em 2008, 80% das crianças e adolescentes com DRC-d se concentravam na Europa, Japão, Estados Unidos (EUA) e Canadá. A prevalência em diferentes países, no final deste ano, variava de 18/pmpmfe na Rússia, passando por 65/pmpmfe no Canadá e Austrália, 85/pmpmfe nos EUA, chegando até cerca de 100/pmpmfe na Finlândia, onde a alta incidência de DRC-d em recém-nascidos, produzida pela síndrome nefrótica do tipo finlandês, é responsável por essa elevada taxa^9^.

Dados mais recentes demonstram que, no Brasil, em julho de 2023, havia 157.357 pacientes em diálise, dos quais 0,9% tinham menos de 20 anos, o que correspondia a cerca de 1.416 pacientes nessa faixa etária^10^. A prevalência global foi de 771 por milhão de população, enquanto que, entre crianças e adolescentes, extrapolando-se até os 19 anos completos, foi de cerca de 35/pmpmfe.

Segundo dados da Sociedade Brasileira de Nefrologia (SBN), regional do Maranhão, com exceção de Imperatriz, segunda cidade mais populosa do estado, distante cerca de 630 km da capital, nenhuma outra unidade do interior atende às crianças (<11 anos) com DRC-d. Esse fato pode explicar a alta taxa descrita no presente estudo, observada na capital. Longe de significar um surto de doença renal crônica na população pediátrica de São Luís e representar uma real prevalência, esse achado resulta nada mais do que do efeito da migração de pacientes e famílias para a capital, em busca de atendimento especializado, não disponível na sua região de origem. Dos 32 pacientes em TRS avaliados no ano de 2023, somente quatro eram residentes em São Luís, o que representaria uma prevalência real de cerca de 13/pmpmfe, bem semelhante à observada por outros autores na região Nordeste do país^3^.

Dentre a população descrita no presente estudo, a maioria era do sexo masculino (56,3%), assim como já demonstrado em outros levantamentos epidemiológicos que abordaram a mesma faixa etária, o que não foi surpreendente^3,7^. Algumas anomalias congênitas do trato urinário têm uma prevalência maior em meninos, como as displasias renais e a estenose da junção pielo-ureteral, além da válvula de uretra posterior, que é exclusiva do sexo masculino^1^.

A idade média da amostra populacional estudada foi de cerca de 11 anos, aproximando-se da observada em outros estudos nacionais que também avaliaram o tratamento dialítico crônico entre crianças e adolescentes^3,4,5^. Nos EUA e na Europa ocidental, a incidência de DRC-d é duas vezes maior em adolescentes (11–19 anos) do que em crianças (≤10 anos)^9^. Como no presente trabalho avaliou-se apenas a prevalência desta condição, não foi possível determinar em qual faixa etária pediátrica ela é mais incidente no nosso meio, considerando que o tempo médio de diálise no grupo avaliado foi de 2 anos e 7 meses, variando de 2 semanas a 98 meses.

Em relação à etnia, a maioria (71,8%) era declarada como não branca nos prontuários médicos pesquisados. Nos EUA, crianças afro-americanas têm duas vezes mais risco de DRC-d do que crianças brancas^11^. Na Austrália e Nova Zelândia, a incidência geral de doença renal é maior nos ditos aborígenes do que entre o restante da população na faixa etária pediátrica, embora a incidência de diálise seja maior somente entre aqueles nativos com idade superior a 15 anos. No Reino Unido, em 2008, a prevalência e a incidência dessa condição observadas entre crianças que emigraram do sudeste asiático eram de 2,5 e 1,5 vezes, respectivamente, maiores do que as observadas na população branca entre 0 e 15 anos^9^. Uma das explicações possíveis para esses achados seria que a desigualdade social e econômica observada entre esses grupos étnicos poderia aumentar a sua vulnerabilidade biológica, além de um possível menor acesso ao diagnóstico e tratamento precoces.

No Brasil, por outro lado, Konstantyner et al.^3^ observaram que o Sul e o Sudeste eram as regiões de maior prevalência e incidência de DRC-d, o que é contraditório ao resultado esperado, já que no país são as regiões de maior prevalência de crianças brancas em TRS. Mais do que fatores sociais ou biológicos, este achado está associado principalmente às características raciais dessa faixa territorial, colonizada ao longo do século passado por uma população considerável de imigrantes europeus e demograficamente com menor proporção de não brancos, segundo o IBGE^8^. Além disso, nesse mesmo estudo multicêntrico citado, houve maior prevalência de não brancos na região Nordeste, Norte e Centro-Oeste, faixas territoriais de menor incidência e prevalência de DRC-d. Esse achado demonstra, aparentemente, apenas a diversificação racial da população pediátrica em diálise crônica de acordo com fatores regionais, mais do que uma relação entre causa e efeito. No estado do Maranhão, por exemplo, 79% da população se autodeclara parda, negra ou indígena e apenas 20,1% se considera branca^8^, dados bem próximos aos observados no presente trabalho em relação à população dialítica pediátrica estudada.

Ao avaliar a frequência escolar das crianças e adolescentes em tratamento dialítico em São Luís, a maioria apresentava-se regular e na classe adequada (90,6%). Tais achados não correspondem ao observado por outros autores em outras instituições, que observaram uma elevada proporção dessa população ausente dos estudos, com atraso escolar ou excesso de faltas^12,13,14^. As motivações são diversas. A fadiga e a dor restringem as atividades diárias. Além disso, as hospitalizações e consultas médicas frequentes têm um efeito cumulativo em relação ao tempo do paciente, monopolizando suas vidas e restringindo sua capacidade de frequentar a escola e participar de atividades extracurriculares. Isso contribui para pior desempenho acadêmico, isolamento social, baixa autoestima, além de menor nível educacional e de empregabilidade na idade adulta^12^.

Atribui-se essa diferença positiva observada no estudo aqui apresentado à presença de um projeto pedagógico de uma escola municipal local, implantado por meio de um convênio entre o HU-UFMA e a Secretaria de Educação da capital. Esse projeto fornece apoio educacional para as disciplinas básicas durante o horário de realização do tratamento, uma iniciativa já consolidada entre crianças em atendimento oncológico no Brasil, mas considerada incomum entre aquelas em TRS.

Mais da metade dos pacientes (68,75%) avaliados no presente estudo apresentava renda familiar (RF) de até um salário mínimo (SM). Duas famílias não apresentavam renda no ano de 2023, uma vez que mãe e filho se encontravam em uma casa de apoio do seu município, situada na capital, e ainda não possuíam acesso ao benefício de prestação continuada fornecido pelo Governo Federal. Apenas um grupo familiar apresentava RF entre dois e três SM. Outros estudos nacionais observaram prevalências que variaram de cerca de 39% a 87% de RF menor que um SM entre crianças e adolescentes dialíticos^15,16^.

Os dados aqui apresentados demonstram, portanto, que a maioria dos pacientes atendidos na capital pertence a famílias de baixa renda. Isso reflete as características socioeconômicas da população atendida, que, na quase totalidade, é procedente do interior e demonstra alta vulnerabilidade financeira e total dependência do suporte governamental para a realização do tratamento. A baixa renda familiar reforça também a limitação no acesso a recursos adicionais que poderiam otimizar o cuidado.

Dentre a população estudada, todos realizavam HD como tipo de TRS no período avaliado, uma vez que o HU-UFMA não realiza DP desde o ano de 2020. A HD como TRS exclusiva da amostra populacional de um hospital de referência em diálise pediátrica no estado leva à reflexão acerca do motivo da falta de disponibilidade do método peritoneal na região. Esse cenário destaca a necessidade de ampliar o acesso a essa modalidade de TRS, considerada mais adequada para o manejo pediátrico. As vantagens significativas para a faixa etária em questão incluem melhor preservação da função renal residual e dos acessos vasculares para um futuro transplante; menor impacto no retardo do crescimento e na necessidade de transfusões; ausência de punções dolorosas; eficiência superior do método em crianças, quando comparada aos adultos, devido à maior superfície peritoneal em relação à superfície corpórea; e possibilidade de realização da TRS em ambiente domiciliar, consequentemente promovendo melhor qualidade de vida e maior adesão ao tratamento^2^.

Por esse motivo, portanto, a DP constitui uma opção viável para pacientes do interior, distantes dos centros especializados, evitando deslocamentos longos e mudanças de domicílio, como ocorreu com a população avaliada no presente estudo. Além disso, recém-nascidos e lactentes com poucos meses de idade ou baixo peso, prematuros extremos e aqueles que nascem com problemas congênitos dos rins e do trato urinário, com consequente DRC, têm nesse método sua única possibilidade de sobreviver até um eventual transplante^2^.

Em um estudo patrocinado pela SBN, Palma et al.^17^ avaliaram 60 centros de diálise pediátrica no país e identificaram que 23 deles não mais ofereciam a DP. Dos 37 restantes, em 19 houve necessidade de conversão de DP para HD devido ao desabastecimento de insumos. Os autores discorrem que a menor disponibilidade de DP no Brasil está relacionada aos baixos valores pagos às empresas fornecedoras, à menor remuneração dos profissionais envolvidos e à falta de profissionais habilitados e de material de reposição, o que tem resultado na escolha da HD, mesmo em crianças com indicação de DP^17^.

Os dados aqui apresentados demonstraram que o tempo médio em diálise foi aferido do início da TRS até a data do transplante, para os pacientes submetidos ao procedimento no ano de 2023, ou até 31 de dezembro do mesmo ano, para os demais que permaneceram em terapia dialítica. Esse tempo foi de aproximadamente 31 meses (2 anos e 7 meses), com mediana de 26 meses (2 anos e 2 meses). Na Suíça, essa variável decresceu de 1,6 anos na década de 1980 para 0,34 anos entre 2010 e 2015^18^. No Brasil, um estudo de prevalência realizado em Minas Gerais, em 2006, demonstrou média de tempo em TRS pediátrica de 2,8 anos (± 2,2 anos)^19^, semelhante à casuística aqui descrita.

O menor tempo de espera até o transplante observado nos levantamentos epidemiológicos de outros países talvez reflita melhores políticas de alocação de órgãos para esse grupo de pacientes. Tal disparidade também pode estar relacionada às diferenças nos protocolos de manejo da DRC-d entre diferentes regiões e à maior disponibilidade de doadores, que impactam diretamente no tempo em que crianças e adolescentes permanecem em TRS. Dentre as 32 crianças e adolescentes em diálise incluídos neste estudo, houve nove transplantes renais no ano de 2023 (28,1%), todos com doadores falecidos. Entre aqueles que não foram transplantados, sete estavam listados em fila de espera e 16 não. Destes, a maioria possuía indicação para essa modalidade de tratamento e estava aguardando apenas a entrada no processo de inscrição, o que provavelmente indica a necessidade de criar estratégias no serviço para melhor direcionamento dos pacientes com vistas ao acesso mais rápido à fila única.

Os dados aqui apresentados também demonstraram que, no final do ano de 2023 ou até a data do transplante, entre aqueles que o realizaram, o acesso vascular predominante foi a FAV (56,3%), seguida pelos cateteres para HD de longa (31,2%) e de curta (12,5%) permanência.

No passado recente, talvez pela facilidade e conveniência dos cateteres venosos profundos (CVCs), as FAVs não foram muito utilizadas em crianças. Dos 870 acessos implantados em 552 pacientes, seguidos pelo Registro da Rede Internacional de Hemodiálise Pediátrica (IPHN), cerca de 72% eram CVCs. Dificuldades técnicas para a confecção e maturação, inexperiência da enfermagem em diálise pediátrica na punção de veias arterializadas de pequeno calibre e preocupação dos pais em relação à dor resultaram na relutância em considerar a FAV como primeira opção^20,21^. As evidências do benefício desse tipo de acesso em comparação aos CVCs em programas de diálises de adultos já são conhecidas há algum tempo. Estudos mais recentes na faixa etária pediátrica também demonstraram resultados semelhantes: diálise mais eficiente, menor taxa de infecção e menor necessidade de troca de acessos e, consequentemente, menores taxas de hospitalização^20,21^.

Ainda sobre acessos vasculares, dados do USRDS (*United States Renal Data System*) demonstraram uma taxa de 76% de CVCs na população pediátrica em TRS naquele país em 2024^22^, quase o dobro dos índices observados em São Luís. Tais resultados poderiam ser explicados, provavelmente, por uma perspectiva maior de transplante renal a curto prazo entre as crianças norte-americanas do que na população aqui avaliada, privilegiando-se a confecção de FAV naquele país apenas para aquelas que, por algum motivo, não teriam um horizonte definido para deixar a HD. Contrapondo-se a essa hipótese, em uma publicação do mesmo ano que avaliou os dados do *Global Kidney Health Atlas* da Sociedade Internacional de Nefrologia (ISN-GKHA), observou-se que, em cerca de 25% dos centros avaliados, mais da metade dos pacientes iniciou HD com FAV^23^. Esse achado foi observado especialmente nos países desenvolvidos, nos quais tradicionalmente as crianças dialíticas têm menor tempo de espera para o transplante renal, semelhante ao que ocorre nos Estados Unidos.

Cerca de um terço das crianças aqui avaliadas não tiveram a etiologia da DRC-d identificada. Esse dado é semelhante ao observado no levantamento epidemiológico nacional realizado em 2011 por Konstantyner et al.^3^, cujos resultados foram influenciados, em relação a essa variável, pelos altos índices de ausência de diagnóstico da doença de base na população pediátrica do Norte e Nordeste naquele período. A avaliação tardia da doença, atribuída à dificuldade de acesso aos grandes centros e à escassez de profissionais especializados para o diagnóstico e manejo da doença renal na infância, pode ser responsável por esses achados. Por exemplo, entre a população pediátrica em diálise em São Luís, no ano de 2023, como já descrito, cerca de 90% eram provenientes do interior do estado, que possui uma das menores taxas de razão médico/paciente e de Índice de Desenvolvimento Humano do país^24^.

Na capital do estado, as CAKUTs corresponderam a 25% dos casos, sendo a VUP a mais frequente. Dados do *North American Pediatric Renal Trials and Collaborative Studies* (NAPRTCS), que recebem informações de nefrologia pediátrica de mais de 7.000 crianças e adolescentes dos EUA, identificaram o conjunto formado por CAKUTs (48%) e nefropatias hereditárias (10%) como a etiologia mais comum, seguido pelas GNCs (14%). Nesse registro, como não poderia deixar de ser, a distribuição das causas variou com a idade. As CAKUTs predominaram entre os mais jovens, assim como as GNCs predominaram entre os adolescentes^25^. Da mesma forma, entre as crianças dialíticas em São Luís em 2023, a média de idade dos pacientes com CAKUT foi de 7,6 anos (± 2,97), enquanto entre os com diagnóstico de GNC foi de 11,5 anos (± 3,3).

Dados semelhantes foram observados na população europeia, a partir de levantamentos epidemiológicos da Itália e da Bélgica. As proporções de CAKUTs (58–59%) e nefropatias hereditárias (15–19%) foram ligeiramente maiores, enquanto que as de GNCs foram um pouco menores (5–7%), quando comparadas ao banco de dados do NAPRTCS^26,27^. Por outro lado, descreve-se uma elevada prevalência de doenças genéticas no Oriente Médio, o que se atribui às altas taxas de casamentos consanguíneos. Da mesma forma peculiar, as GNCs são a principal causa de DRC-d na faixa etária pediátrica em diversos estudos envolvendo crianças da Índia, Sudeste Asiático e África Subsaariana, com uma prevalência variando entre 30% e 60%. Esses achados podem estar relacionados à elevada prevalência de infecções virais, bacterianas e parasitárias nessas regiões^9^. Nessas localidades especificamente, pode-se também especular que uma esperada dificuldade de acesso das crianças pequenas à TRS e o consequente óbito precoce poderiam desviar a estatística para as GNCs, que caracteristicamente incidem nas faixas etárias pediátricas mais elevadas.

Os dados aqui apresentados também demonstraram acesso quase universal das crianças e adolescentes em diálise na capital aos agentes estimuladores da eritropoiese e aos medicamentos para prevenção e tratamento do DMO, todos fornecidos gratuitamente pelo Sistema Único de Saúde (SUS) no país. Após preenchimento de receitas, laudos e termos de consentimento, o próprio serviço de nefrologia pediátrica do HU-UFMA/Rede EBSERH, por meio de sua farmácia, busca as medicações e as fornece às famílias para uso domiciliar, para o manejo dos DMO. Além disso, o serviço propicia o armazenamento e a administração hospitalar da eritropoietina alfa recombinante, para o controle da anemia.

Apesar da facilidade para obtenção dos medicamentos citados, apenas 43% das crianças avaliadas no presente estudo atingiram a meta de PTH (<589 pg/mL) preconizada pelo KDIGO 2017^7^. O nível mediano desse hormônio entre as 32 crianças e adolescentes aqui estudados foi de cerca de 650 pg/mL. Para título de comparação, no último Censo Brasileiro de Diálise de 2023, que abrangeu uma população predominantemente adulta, uma porcentagem cerca de duas vezes maior (81%) dos pacientes atingiu a meta de PTH (<600 pg/mL) entre as clínicas que enviaram os dados para a publicação^10^.

Borzych e Rees^28^, por sua vez, ao avaliarem dados de 890 crianças e adolescentes em diálise peritoneal de 24 países, observaram que a maioria (70%) delas estava com o PTH acima da meta adotada (<300 pg/mL), utilizando como parâmetro as recomendações do KDOQI (*Kidney Disease Outcomes Quality Initiative*) de 2005^28,29^. Valores séricos baixos de cálcio e de bicarbonato, valores elevados de fósforo, sexo feminino e maior tempo em diálise foram fatores de risco independentes para o mau controle do hiperparatireoidismo naquela população^28^. Outros estudos também identificaram as doenças renais túbulo-intersticiais e a deficiência de 25-OH vitamina D como variáveis relacionadas às formas mais graves desse tipo de DMO nessa faixa etária^29^. Schramm et al.^4^, ao avaliarem 35 crianças e adolescentes dialíticos em Manaus, capital do Amazonas, Brasil, entre junho de 2018 e abril de 2019, observaram hipocalcemia em 82,8% dos pacientes, hiperfosfatemia em 57,2% e valores de PTH acima da meta em 48,6%^4^.

No presente estudo, ao contrário do que se poderia esperar com base nos altos índices de valores de PTH observados fora da meta, a maioria das crianças e adolescentes apresentava valores médios de cálcio e fósforo dentro da faixa recomendada. Quanto à 25-OH vitamina D e aos níveis de bicarbonato, no ano de 2023, que foi o período da pesquisa, ainda não era rotina a dosagem regular desses marcadores no serviço, não sendo possível, portanto, identificar se eles tiveram algum impacto nos resultados aqui descritos.

Ainda não se sabe ao certo se, entre crianças e adolescentes, o hiperparatireoidismo na DRC tende a ser de mais difícil controle do que entre os adultos, mas torna-se particularmente preocupante devido ao seu impacto irreversível no crescimento e no desenvolvimento ósseo na população pediátrica.

A maioria (81,2%) das crianças e adolescentes avaliados (26/32) utilizava EPO para o tratamento da anemia da DRC e, a despeito disso, apenas 32% atingiram a meta média esperada (Hb ≥11 g/dL) ao final do período do estudo. Essa taxa foi inferior à observada no último Censo Brasileiro de Diálise da SBN (70%), ressalvando-se, contudo, que, nessa casuística, foi utilizado como parâmetro um limite de Hb >10 g/dL e que os pacientes eram quase todos adultos^10^. No estado do Amazonas, no estudo de Schramm et al.^4^ previamente citado, observou-se anemia em 80% das crianças e adolescentes, e todos utilizavam eritropoietina alfa recombinante. Os valores de ferritina e ISAT estavam fora da faixa esperada em somente 26% e 28%, respectivamente. Deficiência absoluta de ferro foi identificada em 17% dos pacientes e funcional em 11%, na mesma publicação^4^. Khalil et al.^30^, ao acompanharem por 18 meses uma coorte de 78 crianças e adolescentes em hemodiálise, observaram que, ao final do período de seguimento, apenas 29,3% dos pacientes atingiram a meta de Hb, 50% a de ISAT e 11,9% de ferritina, preconizadas pelo KDIGO^30^.

No presente trabalho, 80,6% e 77,4%, respectivamente, da população pediátrica dialítica avaliada atingiram a média anual recomendada para os valores de ferritina e ISAT, principais variáveis relacionadas à resposta inadequada à ação da EPO^6^. Entretanto, convém lembrar que, além da ferropenia absoluta ou relativa, outros fatores podem estar relacionados à baixa resposta aos agentes estimuladores da eritropoiese nessa população, como tumores malignos, infecções e doenças inflamatórias crônicas, deficiências vitamínicas, hiperparatireoidismo e diálise inadequada^31^. Essas variáveis não foram todas exploradas no presente estudo mas podem ter contribuído para a baixa meta de Hb atingida na casuística aqui apresentada.

## Conclusões

Dessa forma, com base nos resultados aqui apresentados, conclui-se que a população de crianças e adolescentes em diálise em São Luís, capital do estado do Maranhão, no ano de 2023, tinha uma média de idade de 11 anos e cerca de 2 anos e 7 meses em TRS. Era predominantemente não branca, de baixa renda familiar, com discreta maioria do sexo masculino, mais de 90% procedentes do interior, todos tratados por HD, uma vez que a DP não era disponível no HU-UFMA, hospital que centralizava a TRS pediátrica na capital. Ao final do período de estudo, a maioria apresentava a FAV como acesso, tinha frequência escolar regular, praticamente sem ausências, e possuía acesso quase universal às medicações para tratamento e prevenção da anemia e do DMO. Ainda assim, menos da metade atingiu a meta da média adequada para o PTH e para a Hb. Mais de um terço da população aqui avaliada não teve sua doença de base identificada, o que pode refletir a falta de acompanhamento prévio, provavelmente por dificuldade de acesso ao especialista antes do início da TRS. Um número significativo de crianças e adolescentes foi transplantado durante o período avaliado; contudo, dentre aqueles que não o fizeram, mais da metade tinha condições clínicas e sociais para realizá-lo, mas, por algum motivo, ainda não estava inscrita em fila de espera. Esse cenário demonstra a necessidade de implementar melhorias no acesso a essa outra forma de TRS, que reconhecidamente oferece maior sobrevida e melhor qualidade de vida, especialmente nessa faixa etária.

## Data Availability

Todo o conjunto de dados que dá suporte aos resultados deste estudo está disponível mediante solicitação ao autor correspondente [Ricardo Ferreira Santos, ricardonefro12@hotmail.com]. O conjunto de dados não está publicamente disponível por conter informações que comprometem a privacidade dos participantes da pesquisa.
